# Supplementary material for: The impact of mutations on TP53 protein and MicroRNA expression in HNSCC: Novel insights for diagnostic and therapeutic strategies
Source: PLoS One. 2025 May 7;20(5):e0307859. doi: 10.1371/journal.pone.0307859 (PMC12057960; doi:10.1371/journal.pone.0307859)
Supplement: S1 Table — (DOCX) [file pone.0307859.s005.docx]

S1 table :The result of Structural and Function Change in Tp53 using HOPE server

| **Mutations** | **Amino acids properties** | **Location/contacts** | **Effect of variants on the protein** |
| --- | --- | --- | --- |
| G136H | 1- The mutant residue is bigger than the wild-type residue.  2- The residue is located on the surface of the protein, mutation of this residue can disturb interactions with other molecules or other parts of the protein. | The mutation is located in:  1- A stretch of residues annotated in UniProt as a special region: Interaction with CCAR2. The differences in amino acid properties can disturb this region and disturb its function.  2- Near a highly conserved position.  3- Dna-Binding region  4-The wild-type residue forms a hydrogen bond with Alanine at position 276. 3- The size difference between wild-type and mutant residue makes that the new residue is not in the correct position to make the same hydrogen bond as the original wild-type residue did.  5- The mutated residue is not in contact with a metal, however, one of the neighbouring residues does make a metal-contact that might be affected by the mutation in its vicinity. | The mutation effected the structural of the protein and may disturb the function. |
| R273C | 1- The mutant residue is smaller than the wild-type residue.  2- The wild-type residue charge was positive, the mutant residue charge is neutral.  3- The mutant residue is more hydrophobic than the wild-type residue.  4- The mutation will cause an empty space in the core of the protein.  5-The hydrophobicity of the wild-type and mutant residue differs.  6- The mutation will cause loss of hydrogen bonds in the core of the protein and as a result disturb correct folding. | 1- The mutation is located in DNA binding region and A stretch of residues annotated in UniProt as a special region: Interaction with CCAR2. The differences in amino acid properties can disturb this region and disturb its function.  2-The wild-type residue forms a hydrogen bond with Aspartic Acid at position 281.  3-The wild-type residue forms a salt bridge with Aspartic Acid at position 281and Glutamic Acid at position 285  4- The difference in charge will disturb the ionic interaction made by the original, wild-type residue.  5- The size difference between wild-type and mutant residue makes that the new residue is not in the correct position to make the same hydrogen bond as the original wild-type residue did | The mutation effected the structural of the protein and may disturb the function. |
| G105C | 1- The mutant residue is bigger than the wild-type residue.  2-The mutant residue is more hydrophobic than the wild-type residue.  3-The residue is located on the surface of the protein, mutation of this residue can disturb interactions with other molecules or other parts of the protein.  4-The torsion angles for this residue are unusual. only glycine is flexible enough to make these torsion angles, mutation into another residue will force the local backbone into an incorrect conformation and will disturb the local structure. | 1- The mutation is located within a stretch of residues annotated in UniProt as a special region: Interaction with CCAR2. The differences in amino acid properties can disturb this region and disturb its function  2- Dna-Binding Domain | The mutation effected the structural of the protein and may disturb the function. |
| R280G | 1- The mutant residue is smaller than the wild-type residue. This will cause a possible loss of external interactions.  2-The wild-type residue charge was positive, the mutant residue charge is natural. The charge of the wild-type residue is lost by this mutation. This can cause loss of interactions with other molecules.  3-The mutant residue is more hydrophobic than the wild-type residue. | 1-The residue is located in a DNA binding region. The differences in properties between wild-type and mutant residue can easily cause loss of these interactions or disturb the domain which will affect the function of the protein.  2-The wild-type residue forms a hydrogen bond with Aspartic Acid at position 281  3- The wild-type residue forms a salt bridge with Aspartic Acid at position 281  4- The mutant residue is located near a highly conserved position.  5-The size difference between wild-type and mutant residue makes that the new residue is not in the correct position to make the same hydrogen bond as the original wild-type residue did.  6-The difference in hydrophobicity will affect hydrogen bond formation.  7- The difference in charge will disturb the ionic interaction made by the original, wild-type residue. | The mutation effected the structural of the protein and may disturb the function. |
| G266E | 1-The mutant residue is bigger than the wild-type residue. The wild-type residue was buried in the core of the protein. The mutant residue is bigger and probably will not fit.  2-The wild-type residue charge was neutral, the mutant residue charge is negative. The mutant residue introduces a charge in a buried residue which can lead to protein folding problems.  3-The wild-type residue is more hydrophobic than the mutant residue.  4-The torsion angles for this residue are unusual. only glycine is flexible enough to make these torsion angles, mutation into another residue will force the local backbone into an incorrect conformation and will disturb the local structure. | 1-The mutation is located within a stretch of residues annotated in UniProt as a special region: Interaction with CCAR2. The differences in amino acid properties can disturb this region and disturb its function.  2-The wild-type residue is a glycine, the most flexible of all residues. This flexibility might be necessary for the protein's function. Mutation of this glycine can abolish this function.  3-The mutant residue is located near a highly conserved position. | The mutation effected the structural of the protein and may disturb the function. |

* CCAR2: Cell Cycle and Apoptosis Regulator 2
