# Supplementary material for: The impact of mutations on TP53 protein and MicroRNA expression in HNSCC: Novel insights for diagnostic and therapeutic strategies
Source: PLoS One. 2025 May 7;20(5):e0307859. doi: 10.1371/journal.pone.0307859 (PMC12057960; doi:10.1371/journal.pone.0307859)
Supplement: S1 Fig — (DOCX) [file pone.0307859.s006.docx]

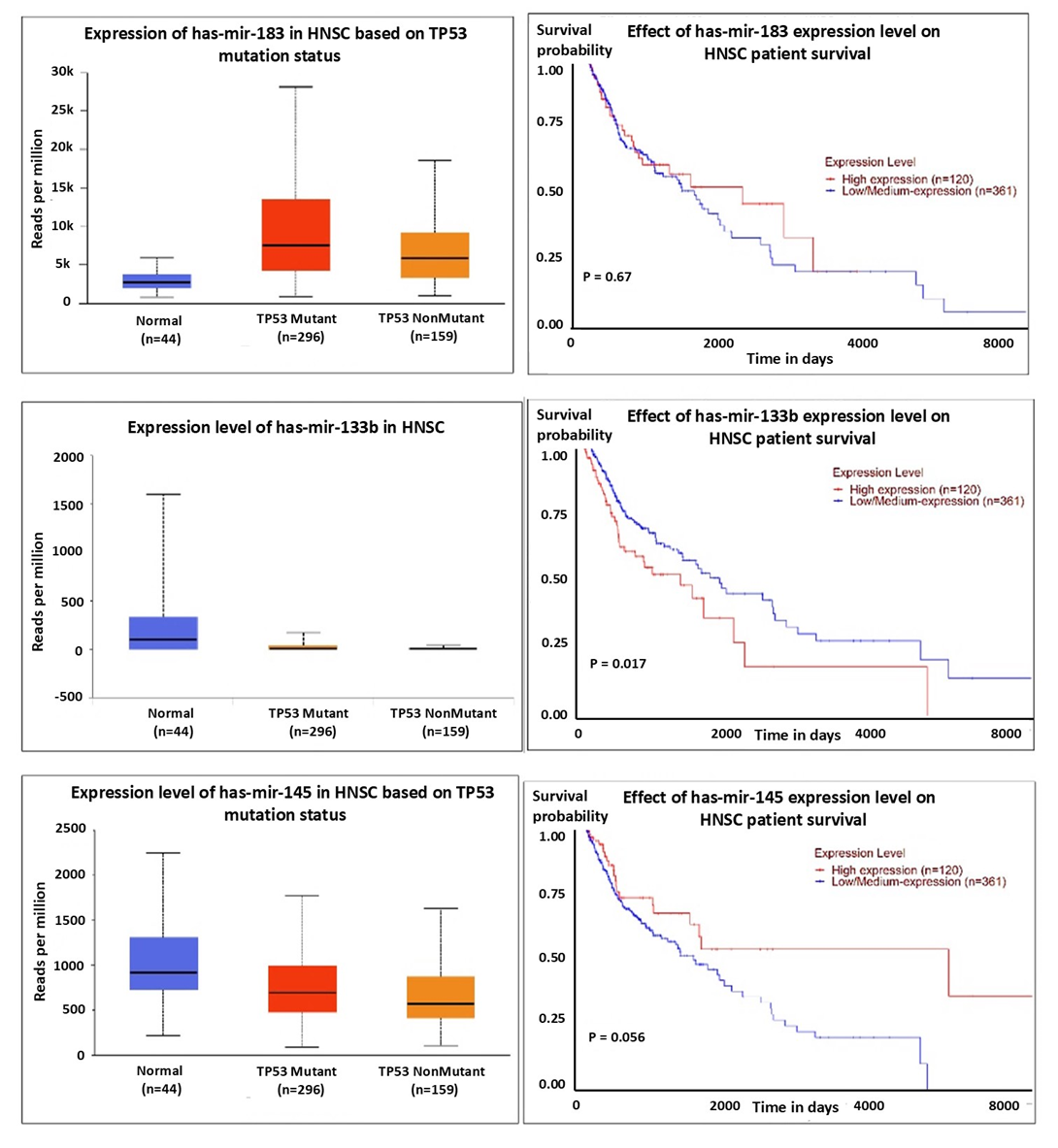


**S1 Fig:** The figure shows the expression of has-mir-183, has-mir-133b, and has-mir-145 in HNSC in TP53 non-mutant, mutant, and normal samples, with the effect of expression of has-mir-183, has-mir-133b, and has-mir-145 on HNSC survival.
