# Supplementary figures and images for: The impact of mutations on TP53 protein and MicroRNA expression in HNSCC: Novel insights for diagnostic and therapeutic strategies
Source: PLoS One. 2025 May 7;20(5):e0307859. doi: 10.1371/journal.pone.0307859 (PMC12057960; doi:10.1371/journal.pone.0307859)

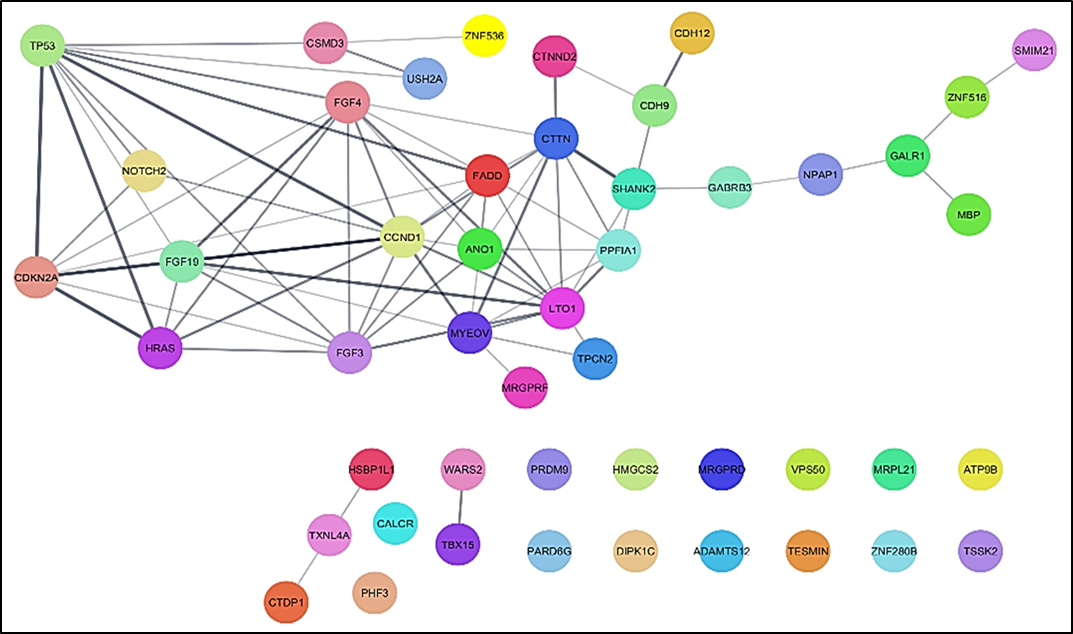


**S2 Fig (Supplementaru Data S3)**: Protein interaction network of TP53 protein.

Supplement: S2 Fig — (DOCX) [file pone.0307859.s007.docx]

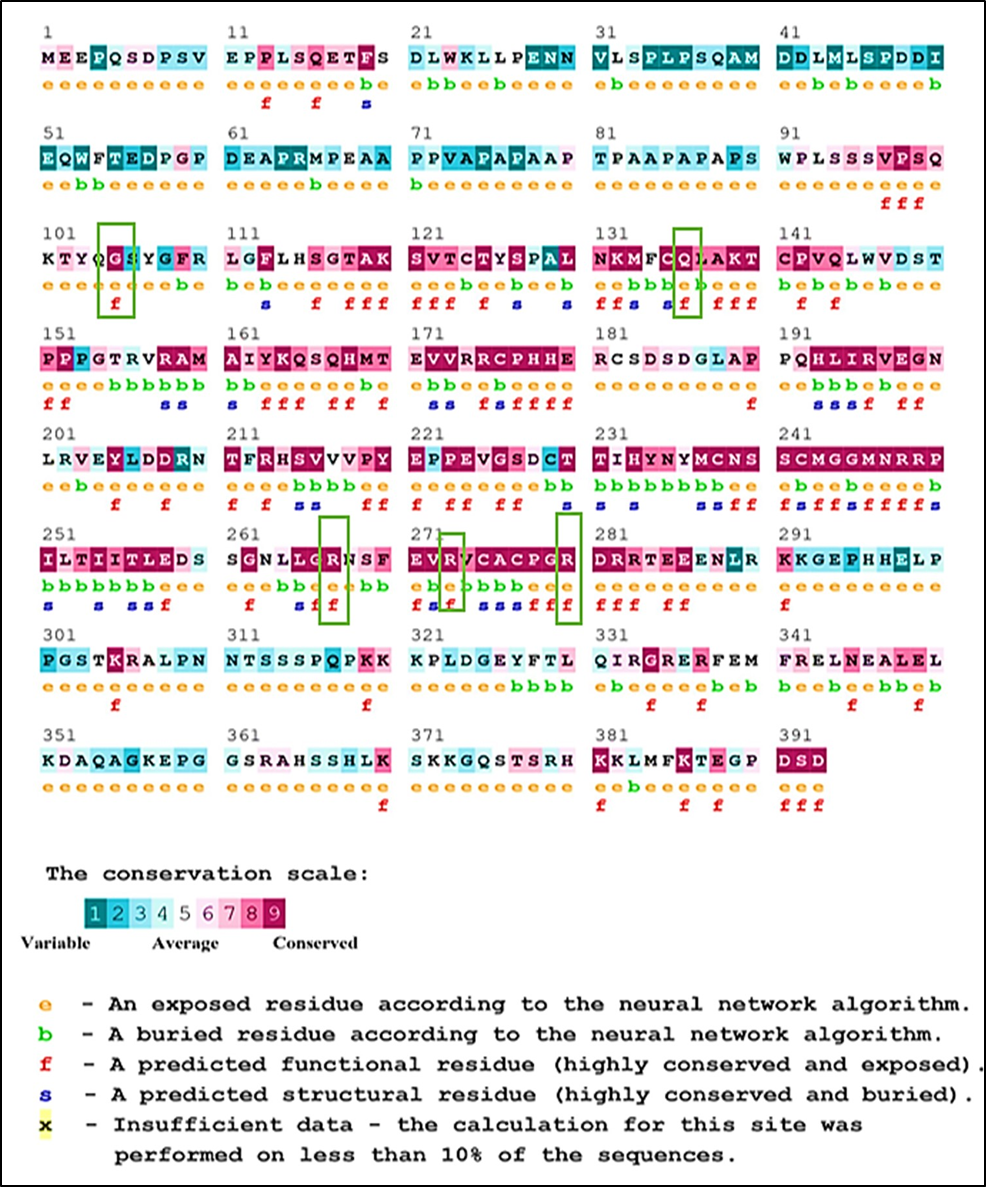


**S3 Fig :** Consurf' analysis of TP53's evolutionary conservation

Supplement: S3 Fig — (DOCX) [file pone.0307859.s008.docx]

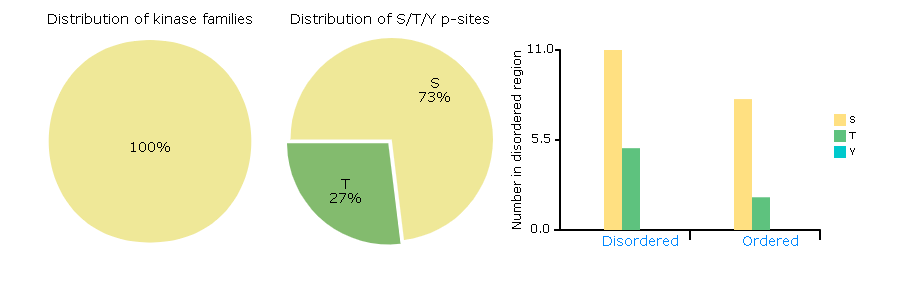


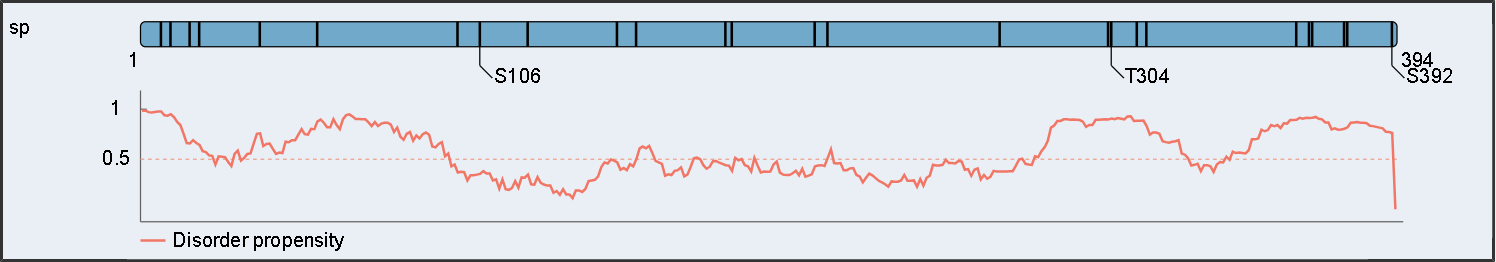


**S4 Fig: Result of the phosphorylation sites**

Supplement: S4 Fig — (DOCX) [file pone.0307859.s009.docx]
