# Supplementary material for: The impact of mutations on TP53 protein and MicroRNA expression in HNSCC: Novel insights for diagnostic and therapeutic strategies
Source: PLoS One. 2025 May 7;20(5):e0307859. doi: 10.1371/journal.pone.0307859 (PMC12057960; doi:10.1371/journal.pone.0307859)
Supplement: S5 Fig — (B) The P53 protein dimer and DNA substrate, colored in yellow and magenta, respectively. The R273C, Q136P, Q136H, and R280G mutations are part of the active site. (DOCX) [file pone.0307859.s010.docx]

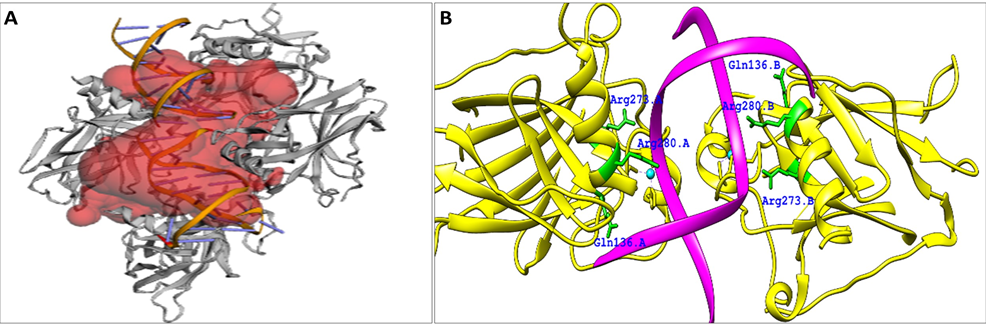


**S5 Fig :**(A)The surface of the TP53 pocket indicated in red ((PDB ID: 2ac0):), computed by CASTp. (B) The P53 protein dimer and DNA substrate were colored in yellow and magent a, respectively the R273C, Q136P, Q136H, and R280G are the parts of active site of protein.
