# Supplementary material for: The impact of mutations on TP53 protein and MicroRNA expression in HNSCC: Novel insights for diagnostic and therapeutic strategies
Source: PLoS One. 2025 May 7;20(5):e0307859. doi: 10.1371/journal.pone.0307859 (PMC12057960; doi:10.1371/journal.pone.0307859)
Supplement: Attachment — Submitted filename: Reviewers Comments.docx [file pone.0307859.s011.docx]

**#Reviewers Comments**

Sofia B Mohamed et al. authored an article titled “**The Impact of Mutations on TP53 Protein and MicroRNA Expression in HNSCC: Novel Insights for Diagnostic and Therapeutic Strategies.**” M.ID: PONE-D-24-29025. The study aimed to explore how mutations affect the structure and function of the TP53 protein and the expression of microRNAs (miRNAs) through computational analysis. Genomic data from patients with HNSCC was sourced from the TCGA database, and the impact of TP53 gene mutations was analyzed using various bioinformatics tools.

The study found that TP53 mutations led to an increase in TP53 expression levels in HNSCC, correlating with a poorer prognosis. Additionally, the authors demonstrated that the expression level of has-mir-133b was significantly reduced in TP53-mutated samples, which had a notable impact on the survival of HNSCC patients. Six mutations—R273C, G105C, G266E, Q136H/P, and R280G—were identified as deleterious, carcinogenic, driver mutations that are highly conserved and exposed. These mutations were located within the P53 domain, and PTM analysis revealed that R280G and R273C are at a methylation site, while R273C, Q136H/P, and R280G are located in the protein pocket. Docking studies indicated that these mutations decreased DNA-binding affinity, with R273C, R280G, G266E, and G105C showing the most significant differences. Molecular dynamics analysis suggested that the R280G, Q136H, and G105C mutations conferred a gain of function by stabilizing the TP53-substrate complex.

The research findings suggest that TP53 mutations influence protein and miRNA expression, as well as the development, survival, and progression of HNSCC patients. Additionally, has-mir-133b could serve as a promising novel biomarker for monitoring HNSCC progression. The authors assert that G105C and Q136H/P, identified as novel mutations, impact the structure and function of proteins involved in HNSCC, making them potential subjects for further investigation, diagnostics, and therapeutic strategies. This research provides new insights into the mechanisms by which these mutations contribute to cancer development.

The work by Sofia B Mohamed et al. is suitable for acceptance, provided the authors address the following major and minor revisions as outlined in the comments.

**Minor comments:**

1. There are some grammatical mistakes and few words in the manuscript are merged, correct them.
2. Most of the figures shown are not clear, please show them in perfect resolution.
3. Conclusion: This study indicated that the mutations in the tp53 protein linked to HNSCC have: Correct TP53, and should be in capital.

**Major comments:**

1. The research begins with a strong introduction, highlighting the significance of TP53 mutations in various human malignancies, particularly HNSCC. However, a brief mention of why TP53 is considered a "tumor suppressor" and its general role in cancer biology could further strengthen the context.
2. Whole work is done and investigated using computational analysis, it would be better and complete if *in vitro*/ *in vivo* studies were also provided in this research for validation.
3. If there is any related in vitro study done so far, please mention and cite.
4. Identifying specific mutations (R273C, G105C, G266E, etc.) as harmful, carcinogenic, and conserved is well-presented. However, it would be beneficial to explain the significance of these terms briefly (e.g., what makes a mutation "deleterious" or "carcinogenic"?).
5. The reduced expression of has-mir-133b in TP53 and its impact on survival is an important finding. It may be useful to elaborate on the role of this specific miRNA in cancer, or why its downregulation is particularly significant.
6. The discovery of G105C and Q136H/P as novel mutations and their implications is a key highlight. Highlighting why these mutations are novel or different from previously known mutations could add more value to the findings.
7. If it can be taken into account as per the methodology used then the research paper is well written and the methodology is fine, though for validation of this work, bioinformatics is not alone enough if the journal doesn’t focus on bioinformatics tools.

Accept the manuscript after the authors have addressed the queries and responded to the comments. It should be accepted for publication, particularly if the journal or issue to which this manuscript was submitted focuses more on computational studies (data is enough and in detail with statistical data in supplementary) than on in vitro research.
