# Supplementary material for: The impact of mutations on TP53 protein and MicroRNA expression in HNSCC: Novel insights for diagnostic and therapeutic strategies
Source: PLoS One. 2025 May 7;20(5):e0307859. doi: 10.1371/journal.pone.0307859 (PMC12057960; doi:10.1371/journal.pone.0307859)
Supplement: Attachment — Submitted filename: Reviewers Comments resubmit.docx [file pone.0307859.s013.docx]

**Editor Comments:**

1. The manuscript contains too many figures, many of which are of poor quality. The clarity and resolution of these figures need significant improvement to ensure that they convey the data accurately.

We take note of the editor’s comments. The number of figures have been reduced to 15. We have also improved the clarity and resolution of the figures as requested.

2. The manuscript employs 100 ns simulations to analyze the structural and dynamic impacts of TP53 mutations. However, this simulation time is insufficient to capture all the relevant conformational changes in the system. I recommend extending the simulation time to at least 200–300 ns.

We have increased the simulation time as requested to 200 ns.

3. The results section contains a substantial amount of information, but it lacks concise explanations.

We have addressed the issue highlighted by adding concise explanations on pages 206-211, 215-217, 219, 221-222, 223-224, 234-240, 295-262, 266-269, 273-277, 282-284, 291-296, 299-303, 309-311, 324-329, 348-351, and 359-369.

4. The results are not interpreted in-depth in the context of HNSCC biology and clinical relevance. Authors should expand the discussion to analyze the biological implications of the mutations, particularly how they may inform diagnostics and treatment strategies in a clinical setting.

We have added more in-depth interpretations of the results in discussion on pages 425-435 and 466-467

5. Survival data appear to be underexplained. Provide more comprehensive statistical methods and interpretations.

Thank you for your feedback on the survival data presented in our study. We recognize the importance of thorough statistical analyses for interpreting such data. However, in this research, we focused solely on the data from the TCGA Pan Cancer Atlas without performing additional statistical analyses. Our objective was to provide a clear overview based on these comprehensive datasets, allowing for straightforward interpretation.

The reasons for not including further statistical analyses are as follows:

- **Focus on Existing Data**: We prioritized utilizing the extensive TCGA datasets to identify patterns between TP53 mutations and hsa-mir-133b expression, enabling readers to directly interpret the data.
- **Clarity and Simplicity**: To avoid complex statistical interpretations, we chose to present the findings in a clear, straightforward manner, emphasizing their biological and clinical relevance.
- **Future Research**: We recognize that additional analyses could deepen our findings and will consider them in future research.

We have added this discussion to better provide context to the readers on page 409-415 of the revised manuscript. We hope this explanation addresses your concerns and is acceptable within the context and scope of what we wanted to achieve for this study.

6. The authors have not fully explored how PTMs impact TP53’s interaction with other proteins or the regulation of its activity.

We have added the following discussion on page 436-446 of the revised manuscript.

**#Reviewers Comments**

**Minor comments:**

1. There are some grammatical mistakes and few words in the manuscript are merged, correct them.

We have proofread the manuscript and corrected grammatical mistakes and other errors.

2. Most of the figures shown are not clear, please show them in perfect resolution.

The resolution of the figures have been improved.

3. Conclusion: This study indicated that the mutations in the tp53 protein linked to HNSCC have: Correct TP53, and should be in capital.

We have corrected this error.

**Major comments:**

1. The research begins with a strong introduction, highlighting the significance of TP53 mutations in various human malignancies, particularly HNSCC. However, a brief mention of why TP53 is considered a "tumor suppressor" and its general role in cancer biology could further strengthen the context.

We have added the following text to further strengthen the context to page 56-62

Whole work is done and investigated using computational analysis, it would be better and complete if *in vitro* / *in vivo* studies were also provided in this research for validation.

Thank you for the valuable feedback regarding the need for in vitro or in vivo studies to complement our computational analysis. We acknowledge that experimental validation is crucial in the field of cancer research.

However, in this study, we focused exclusively on computational analysis to investigate TP53 mutations and their implications for HNSCC using data from the TCGA Pan Cancer Atlas. Our aim was to leverage the extensive genomic datasets available to identify patterns and correlations without conducting laboratory-based experiments.

This work was able to provide a comprehensive analysis of existing datasets to highlight the potential implications of TP53 mutations and has-mir-133b expression on HNSCC progression. We believe that computational methods can offer valuable insights that provide a solid foundation and the theoretical framework for future experimental work. This can lead to substantial cost-saving that will allow for optimization of resources to focus on specific and conclusive experiments that will in turn provide the validation and context, including aspects of understanding the underlying biological mechanisms.

The importance of experimental validation is undeniable, however, the work needed to validate our predictions would be quite extensive and would distract from the main focus of analysing the abundantly available data for formulating the directions of experimental validation. With the context available in the literature, our current computational analysis still provides a meaningful contribution to the understanding of TP53 mutations in HNSCC and serves as a step forward for future experimental validation.”

2. If there is any related in vitro study done so far, please mention and cite.

We have added the discussion below on a related in vitro study to page 508-512 of the revised manuscript.

3. Identifying specific mutations (R273C, G105C, G266E, etc.) as harmful, carcinogenic, and conserved is well-presented. However, it would be beneficial to explain the significance of these terms briefly (e.g., what makes a mutation "deleterious" or "carcinogenic"?).

We have added an explanation on the significance and how the terms can be distinguished below which can be found on page 425-435 of the revised manuscript.

4. The reduced expression of has-mir-133b in TP53 and its impact on survival is an important finding. It may be useful to elaborate on the role of this specific miRNA in cancer, or why its downregulation is particularly significant.

We have elaborated on the role of this specific miRNA in cancer as written below which we have added to page 409 -416.

5. The discovery of G105C and Q136H/P as novel mutations and their implications is a key highlight. Highlighting why these mutations are novel or different from previously known mutations could add more value to the findings.

We have added a discussion on how the G105C and Q136H/P mutations can be distinguished from previously known mutations. The additional discussion provided below can be found on page 513-526 of the revised manuscript.

6. If it can be taken into account as per the methodology used then the research paper is well written and the methodology is fine, though for validation of this work, bioinformatics is not alone enough if the journal doesn’t focus on bioinformatics tools.

Thank you for supportive comments regarding the methodology used and the manuscript in general. To our knowledge, the journal has previously accepted purely computational analyses manuscripts. We have addressed the issue of experimental validation as a response to a previous comment.
