# Supplementary material for: The impact of mutations on TP53 protein and MicroRNA expression in HNSCC: Novel insights for diagnostic and therapeutic strategies
Source: PLoS One. 2025 May 7;20(5):e0307859. doi: 10.1371/journal.pone.0307859 (PMC12057960; doi:10.1371/journal.pone.0307859)
Supplement: Attachment — Submitted filename: Response to Reviewers.docx [file pone.0307859.s014.docx]

**Editor Comments:**

1. The manuscript contains too many figures, many of which are of poor quality. The clarity and resolution of these figures need significant improvement to ensure that they convey the data accurately.

We take note of the editor’s comments. The number of figures have been reduced to 8. We have also improved the clarity and resolution of the figures as requested.

2. The manuscript employs 100 ns simulations to analyze the structural and dynamic impacts of TP53 mutations. However, this simulation time is insufficient to capture all the relevant conformational changes in the system. I recommend extending the simulation time to at least 200–300 ns.

We have increased the simulation time as requested to 200 ns.

3. The results section contains a substantial amount of information, but it lacks concise explanations.

We have addressed the issue highlighted by adding concise explanations on pages 206-211, 215-217, 219, 221-222, 223-224, 234-240, 295-262, 266-269, 273-277, 282-284, 291-296, 299-303, 309-311, 324-329, 348-351, and 359-369.

4. The results are not interpreted in-depth in the context of HNSCC biology and clinical relevance. Authors should expand the discussion to analyze the biological implications of the mutations, particularly how they may inform diagnostics and treatment strategies in a clinical setting.

We have added more in-depth interpretations of the results in discussion on pages 425-435 and 466-467

5. Survival data appear to be underexplained. Provide more comprehensive statistical methods and interpretations.

Thank you for your feedback on the survival data presented in our study. We recognize the importance of thorough statistical analyses for interpreting such data. However, in this research, we focused solely on the data from the TCGA Pan Cancer Atlas without performing additional statistical analyses. Our objective was to provide a clear overview based on these comprehensive datasets, allowing for straightforward interpretation.

The reasons for not including further statistical analyses are as follows:

- Focus on Existing Data: We prioritized utilizing the extensive TCGA datasets to identify patterns between TP53 mutations and hsa-mir-133b expression, enabling readers to directly interpret the data.
- Clarity and Simplicity: To avoid complex statistical interpretations, we chose to present the findings in a clear, straightforward manner, emphasizing their biological and clinical relevance.
- Future Research: We recognize that additional analyses could deepen our findings and will consider them in future research.

We have added this discussion to better provide context to the readers on page 409-415 of the revised manuscript. We hope this explanation addresses your concerns and is acceptable within the context and scope of what we wanted to achieve for this study.

6. The authors have not fully explored how PTMs impact TP53’s interaction with other proteins or the regulation of its activity.

We have added the following discussion on page 436-446 of the revised manuscript.

**Additional Editor Comments:**

I could not find the authors's response to Reviewer 2.

Thank you for your feedback. We would like to clarify that we have addressed all the comments and suggestions provided by **Reviewer 2** in our revised manuscript. If you need further clarification on any specific point, please feel free to let us know, and we would be happy to provide additional details.

Moreover, I noticed significant differences in the MD simulation parameters between the earlier 100 ns simulations and the current 200 ns simulations. Could the authors clarify the source of this inconsistency? Were the simulations reinitiated, or were the previous 100 ns simulations extended? Additionally, what is the origin of the prominent peak observed in the RMSD and Rg of Apo-TP53? Furthermore, why was there a significant difference in the intermolecular hydrogen bonds between the 100 ns and 200 ns trajectories?

Thank you for your questions. Here are the detailed answers:

There is significant differences in the MD simulation parameters between the earlier 100 ns simulations and the current 200 ns simulations. Could the authors clarify the source of this inconsistency?

In the initial submission, the structure coordinates were not optimised before the simulation parameters were calculated (During the protein simulation, the protein moved outside the designated water box, resulting in a truncated structure. This displacement caused the loss of proper spatial constraints, leading to an incomplete or distorted conformation that affected the accuracy of the simulation. In the revised MD simulation, the structures were properly re-centred using Periodic boundary conditions (PBC) to maintain continuity and proper structural representation.

Were the simulations reinitiated, or were the previous 100 ns simulations extended?

The simulations were extended. The 100 ns simulations were not reinitiated; rather, they were extended to 200 ns to allow for more complete equilibration and observation of longer-term dynamics.

What is the origin of the prominent peak observed in the RMSD and Rg of Apo-TP53?

The prominent peak in RMSD and Rg of Apo-TP53 originates from the fact that the TP53 protein was simulated as a dimer in all simulations. However, in the apo structure (without the DNA substrate), the absence of the stabilizing DNA oligomer led to dissociation of the protein dimer, causing significant fluctuations in the RMSD and Rg values as the dimeric interface was destabilized.

Why was there a significant difference in the intermolecular hydrogen bonds between the 100 ns and 200 ns trajectories?

This difference is closely related to the first question with regards to the significant differences in the MD simulation parameters. In the original 100 ns simulation, the structure coordinates were not optimized before calculating the simulation parameters, leading to inaccuracies in the interaction between the mutant TP53 and the DNA oligomer. As a result, the intermolecular hydrogen bonds were not properly quantified.

To ensure the robustness and reproducibility of the findings, I suggest performing MD simulations in triplicate.

Thank you for your valuable suggestion. We appreciate your recommendation to perform MD simulations in triplicate to ensure robustness and reproducibility. However, we believe that extending the simulations to **200 ns** provides sufficient sampling for the analysis, and given the time and computational constraints, performing triplicate simulations may not be necessary for this study. The **200 ns extension** already allowed for comprehensive equilibration and the observation of long-term dynamics, which should be adequate for drawing reliable conclusions. We are confident in the robustness of our findings based on this approach.

**#Reviewers Comments**

I sincerely thank you for taking the time to provide valuable feedback and constructive comments on our manuscript. Your insightful suggestions and observations have been instrumental in improving the quality and clarity of our work.

**Minor comments:**

1. There are some grammatical mistakes and few words in the manuscript are merged, correct them.

We have proofread the manuscript and corrected grammatical mistakes and other errors.

2. Most of the figures shown are not clear, please show them in perfect resolution.

The resolution of the figures have been improved.

3. Conclusion: This study indicated that the mutations in the tp53 protein linked to HNSCC have: Correct TP53, and should be in capital.

We have corrected this error.

**Major comments**:

1. The research begins with a strong introduction, highlighting the significance of TP53 mutations in various human malignancies, particularly HNSCC. However, a brief mention of why TP53 is considered a "tumor suppressor" and its general role in cancer biology could further strengthen the context.

We have added the following text to further strengthen the context to page 56-62

Whole work is done and investigated using computational analysis, it would be better and complete if *in vitro* / *in vivo* studies were also provided in this research for validation.

Thank you for the valuable feedback regarding the need for in vitro or in vivo studies to complement our computational analysis. We acknowledge that experimental validation is crucial in the field of cancer research.

However, in this study, we focused exclusively on computational analysis to investigate TP53 mutations and their implications for HNSCC using data from the TCGA Pan Cancer Atlas. Our aim was to leverage the extensive genomic datasets available to identify patterns and correlations without conducting laboratory-based experiments.

This work was able to provide a comprehensive analysis of existing datasets to highlight the potential implications of TP53 mutations and has-mir-133b expression on HNSCC progression. We believe that computational methods can offer valuable insights that provide a solid foundation and the theoretical framework for future experimental work. This can lead to substantial cost-saving that will allow for optimization of resources to focus on specific and conclusive experiments that will in turn provide the validation and context, including aspects of understanding the underlying biological mechanisms.

The importance of experimental validation is undeniable, however, the work needed to validate our predictions would be quite extensive and would distract from the main focus of analysing the abundantly available data for formulating the directions of experimental validation. With the context available in the literature, our current computational analysis still provides a meaningful contribution to the understanding of TP53 mutations in HNSCC and serves as a step forward for future experimental validation.”

2. If there is any related in vitro study done so far, please mention and cite.

We have added the discussion below on a related in vitro study to page 508-512 of the revised manuscript.

3. Identifying specific mutations (R273C, G105C, G266E, etc.) as harmful, carcinogenic, and conserved is well-presented. However, it would be beneficial to explain the significance of these terms briefly (e.g., what makes a mutation "deleterious" or "carcinogenic"?).

We have added an explanation on the significance and how the terms can be distinguished below which can be found on page 425-435 of the revised manuscript.

4. The reduced expression of has-mir-133b in TP53 and its impact on survival is an important finding. It may be useful to elaborate on the role of this specific miRNA in cancer, or why its downregulation is particularly significant.

We have elaborated on the role of this specific miRNA in cancer as written below which we have added to page 409 -416.

5. The discovery of G105C and Q136H/P as novel mutations and their implications is a key highlight. Highlighting why these mutations are novel or different from previously known mutations could add more value to the findings.

We have added a discussion on how the G105C and Q136H/P mutations can be distinguished from previously known mutations. The additional discussion provided below can be found on page 513-526 of the revised manuscript.

6. If it can be taken into account as per the methodology used then the research paper is well written and the methodology is fine, though for validation of this work, bioinformatics is not alone enough if the journal doesn’t focus on bioinformatics tools.

Thank you for supportive comments regarding the methodology used and the manuscript in general. To our knowledge, the journal has previously accepted purely computational analyses manuscripts. We have addressed the issue of experimental validation as a response to a previous comment.

**Reviewer #2:**

1) The authors must mention the study limitations, strength, novelty, and future prospects of the current study.

Thank you for your valuable feedback. We have carefully reviewed your comments and confirm that the novelty and strengths of the study are explicitly discussed in both the Discussion (525-538), and Conclusion sections (525-538). Moreover, we added the future prospects of this study in discussion section (538-541). Additionally, we have incorporated a dedicated section titled "Limitations" to address the study's limitations, as suggested (564-567).
2) The quality of figure can be improved at a higher resoultion.

Thank you for your feedback. We have already improved the quality of the figures in the revised manuscript by providing higher-resolution images to ensure clarity and better visual representation. If further adjustments are needed, we are happy to make them.
3) The figure legends needs to be elaborated.

We revised and elaborated the legends for Figures 1,2, 3, 5, 6, and 7 to provide more detailed and clear descriptions.

4) There are too many grammatical errors throughout the manuscript. Please proof-read via a native english speaker or a professional english editing software.

Thank you for your feedback. We have proofread the manuscript and corrected grammatical mistakes and other errors.
5) Please mention the thresholds used for PPI network construction.

We have added the thresholds used in the methods section (lines 124-125).

6) Please mention a detailed protocol of how preprocessing of TCGA datasets was performed along with batch-correction and statistical analysis.

As stated in the Methods section of the manuscript, we have already detailed the entire preprocessing process for the TCGA datasets. This includes data collection from the TCGA Data Portal and cBioPortal, followed by gene expression analysis using the UALCAN tool and the TCGA-assembler pipeline (106-121). Regarding batch correction and statistical analysis, we did not perform any specific statistical analysis in this study. The analysis focused on exploring gene expression and mutation data, including TP53 and miRNA expression, without conducting statistical testing. We have clarified these points in the Methods section.
7) What was the need for using GEPIA when the TCGA data was already available. Please justify.

We did not use GEPIA in this study. All analyses related to. GEPIA was not part of the methodology in this research.
8) It would be nice if the authors could validate their findings in an external cohort.
Thank you for the suggestion. In this study, our focus was primarily on analyzing the mutations in TP53 and their impact on gene expression and survival, rather than conducting statistical validation. We acknowledge that validating these findings in an external cohort would strengthen the conclusions. However, this was not the primary aim of the current study. Future work will involve external validation of these findings to further confirm their relevance and applicability in different cohorts.
9) The title of study needs to be written as per the study objectives.

The original title, "The Impact of Mutations on TP53 Protein and MicroRNA Expression in HNSCC: Novel Insights for Diagnostic and Therapeutic Strategies," fits well with the objectives and findings of the study. While the study primarily focuses on the impact of TP53 mutations on gene expression and miRNA regulation in HNSCC, it also explores the potential broader implications for diagnostic and therapeutic strategies.

**References:**

Two references (31, and 32) were added in the reference section as below;

31-	Joerger AC, Fersht AR. The p53 pathway: origins, inactivation in cancer, and emerging therapeutic approaches. Annual review of biochemistry. 2016 Jun 2;85(1):375-404.

32-	Chen X, Zhang T, Su W, Dou Z, Zhao D, Jin X, Lei H, Wang J, Xie X, Cheng B, Li Q. Mutant p53 in cancer: from molecular mechanism to therapeutic modulation. Cell Death & Disease. 2022 Nov 18;13(11):974.
